# Integral inequalities under beta function and preinvex type functions

**DOI:** 10.1186/s40064-016-2165-x

**Published:** 2016-04-26

**Authors:** Izhar Ahmad

**Affiliations:** Department of Mathematics and Statistics, King Fahd University of Petroleum and Minerals, Dhahran, 31261 Saudi Arabia

**Keywords:** Euler beta function, Integral inequality, Holder’s inequality, *P*-preinvex function, 33B15, 26B25, 26D15, 26D10

## Abstract

In the present paper, the notion of P-preinvex function is introduced and new integral inequalities for this kind of function along with beta function are establised. The work extends the results appeared in the literature.

## Background

Convexity plays an important role in economics, management science, engineering, finanace and optimization theory. Many interesting generalizations and extensions of classical convexity have been used in optimization and mathematical inequalities. Hanson (1981) introduced the concept of invexity. These functions were named invex by Craven (1981) and $\eta$-convex by Kaul and Kaur (1980). Weir and Mond (1988) introduced the concept of preinvex function. Later, Mohan and Neogy (1995) presented few properties of preinvex functions. Some refinements of the mathematical inequalities on convex and generalized convex functions have been investigated in Barani et al. (2012), Chalco-Cano et al. (2012), Dragomir (2001), Dragomir and Agarwal (1998), Fok and Vong (2015), Matloka (2014), Muddassar and Bhatti (2013) and Pachpatte (2004).

Let *S* be a nonempty subset of $R^n$ and let $\eta : S \times S \rightarrow R^n.$

### **Definition 1**

The set $S\subseteq R^n$ is said to be invex with respect to $\eta (u, v)$ if for every $u, v \in S$ and $t \in [0, 1],$

$$\begin{aligned} v + t \eta (u,v) \in S. \end{aligned}$$

It is obvious that every convex set is invex with respect to $\eta (u, v) = u - v,$ but there exist invex sets which are not convex (see Mohan and Neogy 1995).

### **Definition 2**

(Weir and Mond 1988) The function $f : S \rightarrow R$ is said to be preinvex on *S* with respect to $\eta (u, v)$, if

$$\begin{aligned} f( v + t \eta (u, v)) \le (1 -t) f(v) + t f(u) \end{aligned}$$

for every $u, v \in S$ and $t \in [0, 1],$

The Gauss–Jacobi type quadrature formula has the following

1 $$\begin{aligned} \int _{a}^{b} (x - a)^p(b - x)^q f(x)dx = {\sum \limits _{k = 0}^\infty } B_{m, k} f(\gamma _k) + R^{\star }_{m} \vert f \vert , \end{aligned}$$

for certain $B_{m, k}, \gamma _k$ and rest $R^{\star }_{m} \vert f \vert$ (see Stancu et al. 2002).

Recently, Liu (2014) obtained several integral inequalities for the left hand side of (1) under the following *P*-convexity:

The function $f : I \rightarrow R$, where $I \subseteq R$ is said to be *P*-convex on a convex set, if

$$\begin{aligned} f(tu + (1 -t)v) \le f(u) + f(v). \end{aligned}$$

for every $u, v \in I$ and $t \in [0, 1].$ For the applications of *P*-convex function and its generalizations, we refer Akdemir and Ozdemir (2010), Barani and Barani (2012), Liu (2013, 2014), Tunc (2013) and Varosanec (2007).

The main purpose of this paper is to introduce the class of *P*-preinvex function and derive new inequalities for the left hand side of (1) under these assumptions. The presented results generalize the results of Liu (2014) and references cited therein.

## New integral inequalities

### **Definition 3**

The function $f : S \rightarrow R$ is said to be *P*-preinvex on *S* with respect to $\eta (u, v)$, if

$$\begin{aligned} f(v + t \eta (u,v)) \le f(u) + f(v). \end{aligned}$$

for every $u, v \in S$ and $t \in [0, 1],$

Note that every P-convex function (Liu 2014) is a *P*-preinvex function with respect to $\eta (u,v) = u - v$ for any $t \in [0, 1].$

### **Lemma 1**

*Let*$f: S= [a, a + \eta (b, a)] \rightarrow R$*be a continous function on the interval of real numbers*$S^0$*(the interior ofS)with*$a < a + \eta (b,a).$*Iffis P-preinvex function on*$[a, a + \eta (b, a)]$, *then for some fixed*$p, q > 0$,

$$\begin{aligned} \int _{a}^{ a + \eta (b, a)} (x -a)^p (a + \eta (b, a) -x)^q f(x) dx = \eta (b, a)^{p + q + 1} \int ^{1}_{0} t^p (1 -t)^q f(a + t\eta (b, a))dt. \end{aligned}$$

### *Proof*

It is easy to observe that

$$\begin{aligned} \int _{a}^{ a + \eta (b, a)} (x -a)^p (a + \eta (b, a) -x)^q f(x) dx & ={} \int ^{1}_{0} (a + t\eta (b, a) -a)^p(a + \eta (b, a) - a- t\eta (b, a))^q\\&\quad \times \, f(a + t\eta (b, a))dt\\& = {} \eta (b, a)^{p + q + 1} \int ^{1}_{0} t^p (1 -t)^q f(a + t\eta (b, a))dt \end{aligned}$$

$$\square$$

The following definition will be used in the sequel.

### **Definition 4**

The beta function is defined for $x, y > 0$ as

$$\begin{aligned} \beta (x, y) = \int ^{1}_{0} t^{x -1} ( 1 - t) ^{y -1} dt. \end{aligned}$$

### **Theorem 1**

*Let*$f: S= [a, a + \eta (b, a)] \rightarrow R$*be a continous function on the interval of real numbers*$S^0$ (*the interior ofS*) *with*$a < a + \eta (b,a).$*If*$\vert f \vert$*is P-preinvex function on*$[a, a + \eta (b, a)]$, *then for some fixed*$p, q > 0$,

$$\begin{aligned} \int ^{ a + \theta (b, a)}_{a} (x -a)^p (a + \eta (b, a) -x)^q f(x) dx \le \eta (b, a)^{p + q + 1} \beta (p + 1, q + 1)(\vert f(a)\vert + \vert f(b)\vert ). \end{aligned}$$

### *Proof*

Since $\vert f \vert$ is *P*-preinvex function on $[a, a + \eta (b, a)]$, we have

$$\begin{aligned} \vert f(a + t\eta (b, a))\vert \le \vert f(a) \vert + \vert f(b) \vert \end{aligned}$$

for all $t \in [0,1].$ By Theorem 1 and *P*-preinvexity of $\vert f \vert$, we get

$$\begin{aligned} \int ^{ a + \eta (b, a)}_{a} (x -a)^p (a + \eta (b, a) -x)^q f(x) dx& = {} \eta (b, a)^{p + q + 1} \int ^{1}_{0} t^p (1 -t)^q \vert f(a + t\eta (b, a)) \vert dt\\&\le \eta (b, a)^{p + q + 1} \int ^{1}_{0} t^p (1 -t)^q (\vert f(a) \vert + \vert f(b) \vert )dt\\& = {} \eta (b, a)^{p + q + 1} \beta (p + 1, q + 1)(\vert f(a)\vert + \vert f(b)\vert )\\&\quad (by\; the\;definition\,4). \end{aligned}$$

### **Theorem 2**

*Let*$f: S= [a, a + \eta (b, a)] \rightarrow R$*be a continous function on the interval of real numbers*$S^0$ (*the interior ofS*) *with*$a < a + \eta (b,a).$*If*$\vert f \vert ^{\frac{k}{k -1}}$*is P-preinvex function on*$[a, a + \eta (b, a)]$, *then for some fixed*$p, q > 0$,

$$\begin{aligned}&\int ^{ a + \eta (b, a)}_{a} (x -a)^p (a + \eta (b, a) -x)^q f(x) dx \\&\quad \le \eta (b, a)^{p + q + 1} [\beta (kp + 1, kq + 1)]^{\frac{1}{k}} \left( \vert f(a)\vert ^{\frac{k}{k -1}} + \vert f(b)\vert ^{\frac{k}{k -1}} \right) ^{\frac{k -1}{k}}. \end{aligned}$$

### *Proof*

The *P*-preinvexity of $\vert f \vert ^{\frac{k}{k -1}}$ on $[a, a + \eta (b, a)]$ along with Lemma 1, Definition 4 and H${\ddot{o}}$lder inequality imply that

$$\begin{aligned}&\int ^{ a + \eta (b, a)}_{a} (x -a)^p (a + \eta (b, a) -x)^q f(x) dx\\&\quad \le \eta (b, a)^{p + q + 1} \left[ \int ^{1}_{0} t^{kp} (1 -t)^{kq} \right] ^{\frac{1}{k}} \left[ \int ^{1}_{0} \vert f(a + t\eta (b, a)) \vert ^{\frac{k}{k -1}} dt \right] ^{\frac{k - 1}{k}}\\&\quad \le \eta (b, a)^{p + q + 1} [\beta (kp + 1, kq + 1)]^{\frac{1}{k}} \left[ \int ^{1}_{0}\left( \vert f(a)\vert ^{\frac{k}{k -1}} + \vert f(b)\vert ^{\frac{k}{k -1}} \right) dt \right] ^{\frac{k -1}{k}}\\&\quad = \eta (b, a)^{p + q + 1} [\beta (kp + 1, kq + 1)]^{\frac{1}{k}} \left( \vert f(a)\vert ^{\frac{k}{k -1}} + \vert f(b)\vert ^{\frac{k}{k -1}} \right) ^{\frac{k -1}{k}}.\\ \end{aligned}$$

This completes the proof. $\square$

### **Theorem 3**

*Let*$f: S= [a, a + \eta (b, a)] \rightarrow R$*be a continous function on the interval of real numbers*$S^0$ (*the interior ofS*) *with*$a < a + \eta (b,a).$*If*$\vert f \vert ^l$*is P-preinvex function on*$[a, a + \eta (b, a)]$, *then for some fixed*$p, q > 0$,

$$\begin{aligned} \int ^{ a + \eta (b, a)}_{a} (x -a)^p (a + \eta (b, a) -x)^q f(x) dx \le \eta (b, a)^{p + q + 1} \beta (p + 1, q + 1)\left( \vert f(a)\vert ^l + \vert f(b)\vert ^l \right) . \end{aligned}$$

### *Proof*

The *P*-preinvexity of $\vert f \vert ^{l}$ on $[a, a + \eta (b, a)]$ along with Lemma 1, Definition 4 and H${\ddot{o}}$lder inequality give

$$\begin{aligned}&\int ^{ a + \eta (b, a)}_{a} (x -a)^p (a + \eta (b, a) -x)^q f(x) dx \\&\quad = \eta (b, a)^{p + q + 1} \int ^{1}_{0}\left[ t^{p} (1 -t)^{q} \right] ^{\frac{l-1}{l}} \left[ t^{p} (1 -t)^{q} \right] ^{\frac{l}{l}}f(a + t\eta (b, a)) dt\\&\quad \le \eta (b, a)^{p + q + 1} \left[ \int ^{1}_{0} t^{p} (1 -t)^{q}dt \right] ^{\frac{l-1}{l}} \left[ \int ^{1}_{0} t^{p} (1 -t)^{q} \vert f(a + t\eta (b, a))\vert dt \right] ^{\frac{1}{l}}\\&\quad \le \eta (b, a)^{p + q + 1} [\beta (p + 1, q + 1)]^{\frac{1-l}{l}} \left[ \left( \vert f(a)\vert ^l + \vert f(b)\vert ^l \right) \beta (p + 1, q + 1) \right] ^{\frac{1}{l}}\\&\quad = \eta (b, a)^{p + q + 1} \beta (p + 1, q + 1) \left( \vert f(a)\vert ^l + \vert f(b)\vert ^l \right) ^{\frac{1}{l}}. \end{aligned}$$

This completes the proof. $\square$

## Intergal inequalities involving prequasi-invex

I state the following theorems as the proof follow on the same lines of the theorems of “New integral inequalities” section.

### **Definition 5**

(Pinni 1991) The function $f : S \rightarrow R$ is said to be prequasi-invex on *S* with respect to $\eta (u, v)$, if

$$\begin{aligned} f(t u + ( 1- t)v) \le max {(f(u), f(v))} \end{aligned}$$

for every $u, v \in S$ and $t \in [0, 1].$

### **Theorem 4**

*Let*$f: S= [a, a + \eta (b, a)] \rightarrow R$*be a continous function on the interval of real numbers*$S^0$ (*the interior ofS*) *with*$a < a + \eta (b,a).$*Iffis prequasi-invex function on*$[a, a + \eta (b, a)]$, *then for some fixed*$p, q > 0$

$$\begin{aligned} \int ^{ a + \eta (b, a)}_{a} (x -a)^p (a + \eta (b, a) -x)^q f(x) dx \le \eta (b, a)^{p + q + 1} \beta (p + 1, q + 1) {\mathrm{max}}( f(a), f(b)). \end{aligned}$$

### **Theorem 5**

*Let*$f: S= [a, a + \eta (b, a)] \rightarrow R$*be a continous function on the interval of real numbers*$S^0$ (*the interior ofS*) *with*$a < a + \eta (b,a).$*If*$\vert f \vert$*is prequasi-invex function on*$[a, a + \eta (b, a)]$, *then for some fixed*$p, q > 0$,

$$\begin{aligned} \int ^{ a + \eta (b, a)}_{a} (x -a)^p (a + \eta (b, a) -x)^q f(x) dx \le \eta (b, a)^{p + q + 1} \beta (p + 1, q + 1) max(\vert f(a)\vert , \vert f(b)\vert ). \end{aligned}$$

### **Theorem 6**

*Let*$f: S= [a, a + \eta (b, a)] \rightarrow R$*be a continous function on the interval of real numbers*$S^0$ (*the interior ofS*) *with*$a < a + \eta (b,a).$*If*$\vert f \vert ^{\frac{k}{k -1}}$*is prequasi-invex function on*$[a, a + \eta (b, a)]$, *then for some fixed*$p, q > 0$,

$$\begin{aligned}&\int ^{ a + \eta (b, a)}_{a} (x -a)^p (a + \eta (b, a) -x)^q f(x) dx \\&\quad \le \eta (b, a)^{p + q + 1} [\beta (kp + 1, kq + 1)]^{\frac{1}{k}} max \left( \vert f(a)\vert ^{\frac{k}{k -1}}, \vert f(b)\vert ^{\frac{k}{k -1}} \right) ^{\frac{k -1}{k}}. \end{aligned}$$

### **Theorem 7**

*Let*$f: S= [a, a + \eta (b, a)] \rightarrow R$*be a continous function on the interval of real numbers*$S^0$ (*the interior ofS*) *with*$a < a + \eta (b,a).$*If*$\vert f \vert ^l$*is prequasi-invex function on*$[a, a + \eta (b, a)]$, *then for some fixed*$p, q > 0$,

$$\begin{aligned} \int ^{ a + \eta (b, a)}_{a} (x -a)^p (a + \eta (b, a) -x)^q f(x) dx \le \eta (b, a)^{p + q + 1} \beta (p + 1, q + 1) max\left( \vert f(a)\vert ^l, \vert f(b)\vert ^l \right) . \end{aligned}$$

### *Remark 1*

If $\eta (b, a) = b - a$ in the theorems of “Intergal inequalities involving prequasi-invex” section, then we get the Theorems appeared in Liu (2013).

## Conclusion

In this paper, I have introduced the P-preinvex function and used it along with beta function to establish the new integral type inequalities. I also stated the other integral type inequalities under prequasi-invex function. The presented results may be futher generalized under weaker convexity assumptions.
